# Localized AI for stroke care in LMICs: a framework to overcome structural and diagnostic barriers

**DOI:** 10.3389/fpubh.2026.1859276

**Published:** 2026-06-10

**Authors:** Qing Liu, Xuemei Jia, Yingchun He, Yufeng Hou, Yulin Deng, Zhi Yan

**Affiliations:** 1 Tianfu College of Southwestern University of Finance and Economics, Mianyang, China; 2 School of Medical Technology, Sichuan College of Traditional Chinese Medicine, Mianyang, China; 3 Mianyang 404 Hospital, Mianyang, China; 4 Capital Medical University, Beijing, China

**Keywords:** artificial intelligence, digital public health, edge computing, health equity, stroke care

## Abstract

Low- and middle-income countries (LMICs) bear a disproportionate share of the global stroke burden, driven not only by resource limitations but also by systemic inefficiencies in workforce distribution, diagnostic access, and prehospital care coordination. While advances in artificial intelligence (AI) have demonstrated significant potential in stroke diagnosis and management, many existing solutions remain poorly aligned with the infrastructural and policy realities of LMIC health systems, limiting their scalability and long-term impact. This study presents a comprehensive narrative review of literature published between January 2015 and March 2026, synthesizing evidence across digital health, stroke systems of care, and AI deployment models. We identify three persistent structural barriers—workforce shortages, diagnostic centralization, and fragmented care pathways—that collectively constrain timely intervention in acute stroke. In response, we propose a “Localized AI + Policy” framework that integrates lightweight AI models, edge computing, and federated learning within context-specific health system and governance structures. This approach emphasizes decentralized computation, data sovereignty, and alignment with national health policies, enabling more resilient and scalable deployment of AI in resource-constrained environments. By shifting the focus from technology-centric innovation to system-integrated implementation, this framework highlights a pathway for translating AI advances into sustainable public health impact. The findings underscore the importance of embedding digital health solutions within broader strategies for health system strengthening, universal health coverage, and global health equity.

## 1 Introduction

Globally, stroke remains one of the leading causes of mortality and long-term disability, yet the epidemiological burden is profoundly skewed, with LMICs now bearing an overwhelmingly disproportionate share of both stroke incidence and mortality (1). While acute stroke neurology has achieved unprecedented milestones in high-income countries (HICs)—epitomized by the widespread implementation of rapid reperfusion therapies—translating these life-saving advancements into equitable care within resource-constrained regions remains severely limited (2, 3). This unacceptable disparity is not merely a consequence of absolute resource scarcity; rather, it has become one of the most pressing challenges in the global prevention and control of non-communicable diseases (NCDs). Consequently, this review focuses on the persistent inequities of stroke care in LMICs, directing specific attention to the interplay between deep-seated structural barriers, emerging artificial intelligence (AI) interventions, and their broader policy implications.

Despite a growing body of literature examining global stroke outcomes, there remains a distinct lack of systematic integration and consensus regarding the root causes of these disparities. It is increasingly debated whether the inequality in stroke care stems primarily from technical deficits—such as the profound lack of access to advanced neuroimaging and acute therapies—or if it is more fundamentally driven by deeper institutional, systemic, and policy constraints (4, 5). In particular, research investigating how to synergistically advance technological innovation alongside structural health policy reform remains highly fragmented. This leaves a critical knowledge gap regarding how to implement sustainable, scalable solutions in fragile healthcare ecosystems without exacerbating existing digital divides (6, 7).

Several recent reviews have explored stroke care models or the application of AI in cerebrovascular diseases; however, the majority of these analyses focus predominantly on HICs or concentrate exclusively on the algorithmic performance of the technology itself, largely neglecting the LMIC perspective on structural factors, technological adaptability, and policy pathways (8, 9). Recently, AI technologies have achieved pivotal breakthroughs in medical imaging, telemedicine, and grassroots clinical support—specifically through localized paradigms such as edge computing and federated learning, which explicitly circumvent traditional infrastructure limits (10, 11). Concurrently, digital health governance frameworks and national health system reforms have undergone significant evolution, driven by global initiatives advocating for standardized digital architectures and health equity (12). These converging technical and political shifts necessitate a renewed, comprehensive assessment of the field.

Therefore, this comprehensive review aims to integrate the latest evidence to redefine stroke inequality in LMICs through a combined structural and policy lens. Specifically, it examines how systemic constraints—including workforce limitations, restricted access to diagnostic imaging, and fragmented prehospital systems—translate into distinct but interrelated delays across the stroke care pathway. These delays collectively drive what we conceptualize as “temporal compression,” namely the narrowing or loss of the therapeutic window for acute stroke interventions.

**Figure 1:**
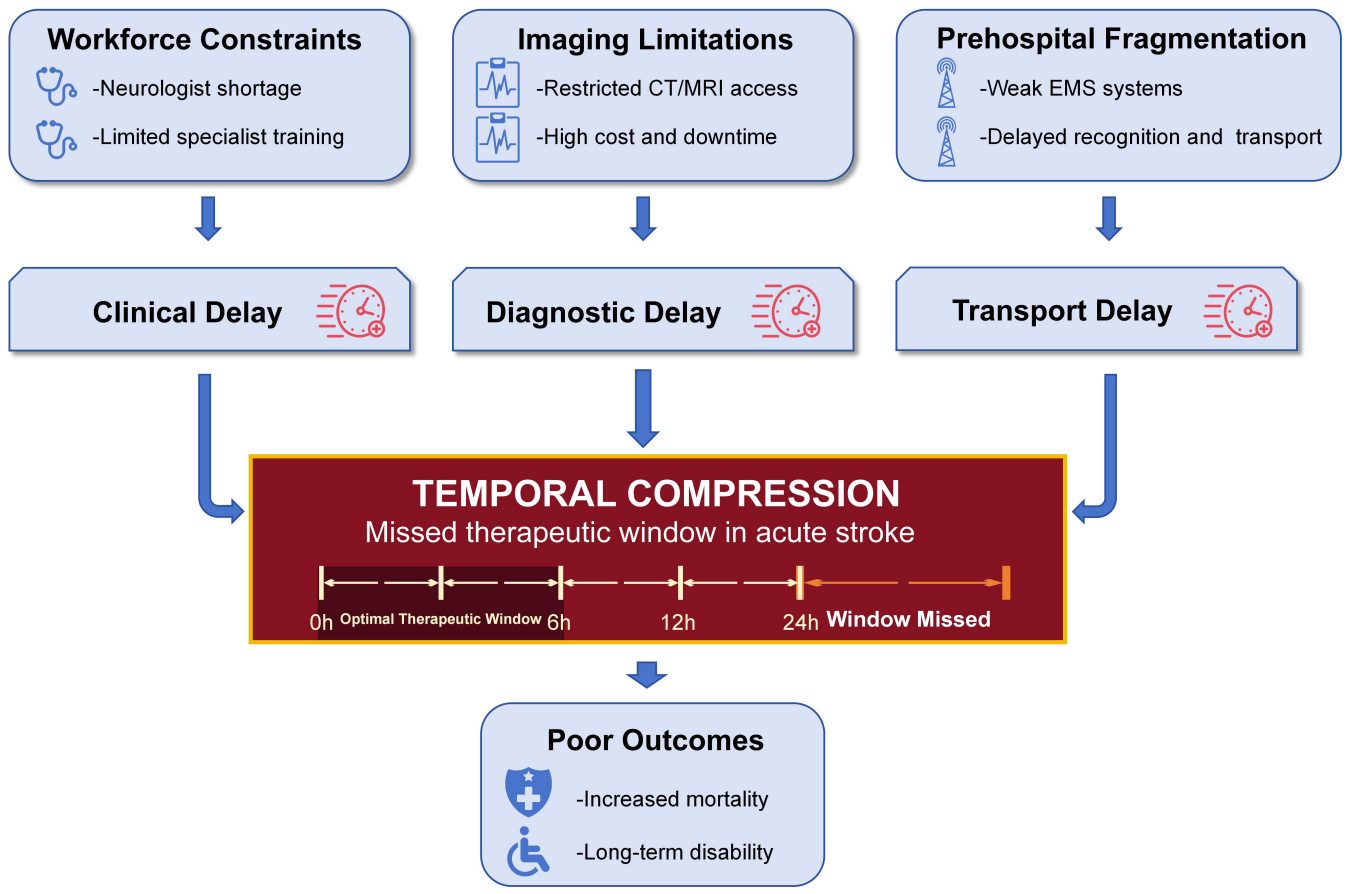
Systemic barriers driving temporal compression in stroke care in low- and middle-income countries (LMICs). Workforce limitations, restricted access to diagnostic imaging, and fragmented prehospital systems contribute to clinical, diagnostic, and transport delays. These delays converge to produce “temporal compression,” defined as the narrowing or loss of the therapeutic window for acute stroke interventions. Note on timeline: The timeline reflects the 2019 AHA/ASA Acute Ischemic Stroke Guidelines, which establish a standard 0-to-6-h window for mechanical thrombectomy relying on non-contrast CT (NCCT) and CTA, and an extended 6-to-24-h window for highly selected patients. However, because the 6-to-24-h extended window strictly requires advanced perfusion imaging (CTP or DW-MRI) to assess the infarct core and penumbra mismatch, the lack of such advanced infrastructure in many LMIC facilities effectively compresses their viable operational window to the standard 0-to-6 h, resulting in missed therapeutic opportunities for late-arriving patients.

To provide a structured overview of this problem architecture, Figure 1 illustrates the convergence of these systemic barriers into temporal compression and their downstream impact on clinical outcomes. Building on this foundation, the subsequent sections evaluate the real-world potential of “Localized AI” solutions—technologies explicitly engineered for resource-constrained environments—and propose an actionable policy-integrated framework to guide equitable and resilient stroke care delivery.

## 2 Methods

### 2.1 Overview

This study was conducted as a structured narrative review to synthesize heterogeneous evidence on artificial intelligence (AI)-enabled stroke care in LMICs. A narrative design was selected because the relevant literature spans multiple disciplines, including digital health, acute stroke systems of care, implementation science, and health policy. Rather than assuming methodological homogeneity, this review was designed to preserve interpretive depth while enabling structured charting of evidence across diverse study designs and evidence types. Accordingly, the included literature was summarized through two complementary descriptive evidence maps: one focused on empirical, diagnostic validation, and implementation studies (Supplementary Table S1), another focused on supporting contextual literature, including review articles, policy analyses, standards documents, and methodological papers (Supplementary Table S2).

### 2.2 Literature search strategy

To ensure methodological transparency and consistency with the PRISMA extension for Scoping Reviews (PRISMA-ScR), a systematic literature search was conducted across five electronic databases: PubMed, IEEE Xplore and Google Scholar. The search strategy covered the period from January 2015 to March 2026. This timeframe was chosen to capture the development of post-2015 implementation science relevant to LMIC health systems, together with the rapid evolution of decentralized AI, edge computing, federated learning, and digital health governance frameworks.

**Figure 2:**
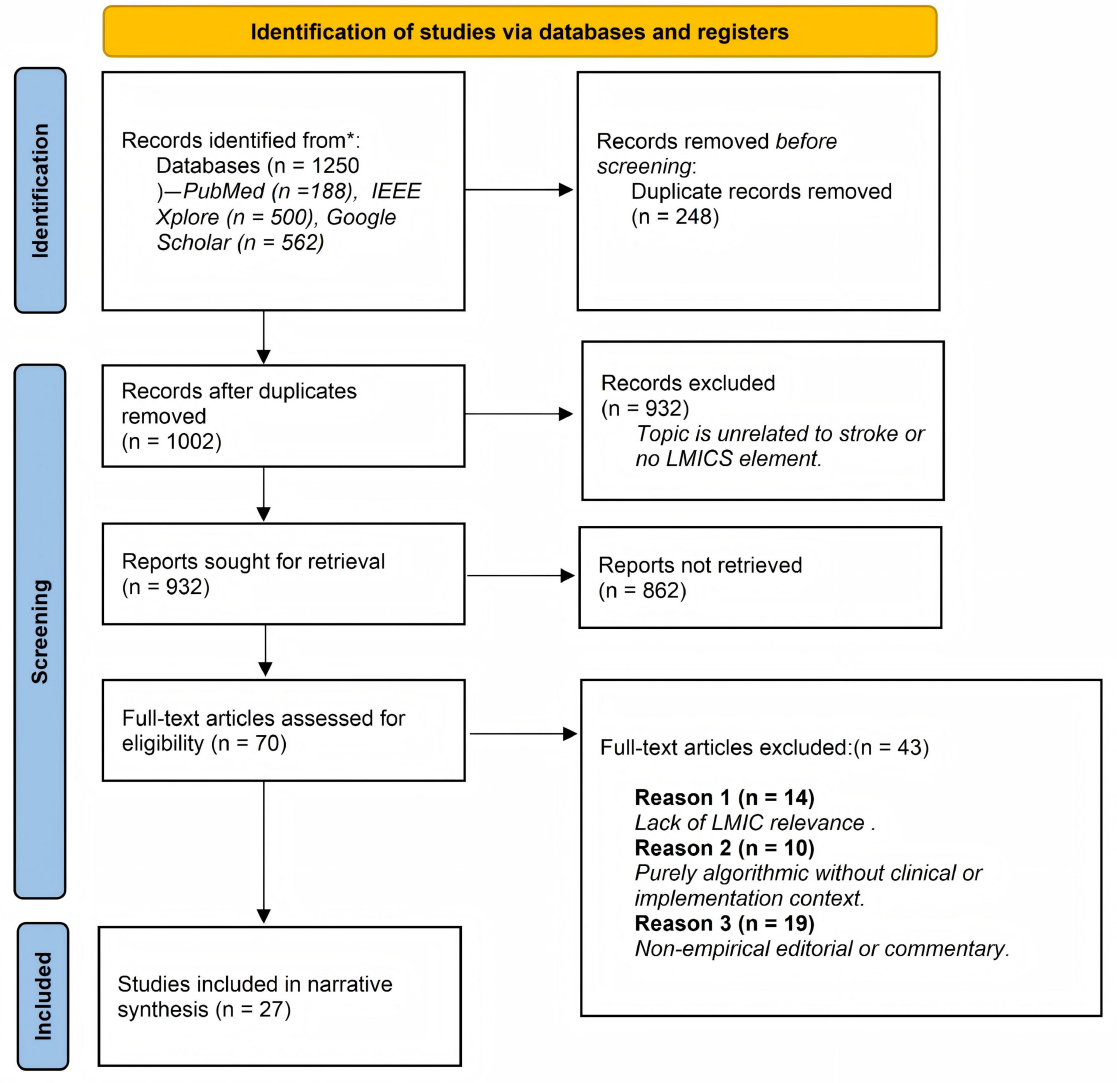
PRISMA 2020 flow diagram illustrating the number of records identified, screened, and included in the narrative synthesis. LMICs, low- and middle-income countries.

The study selection process and categorized reasons for exclusion are detailed in the PRISMA-ScR flow diagram (Figure 2). Database-specific search strings are detailed in Supplementary material S1.

In addition to peer-reviewed literature, selected gray literature—including policy reports (e.g., WHO IGAP) and global health publications—was reviewed to capture system-level perspectives that are not consistently represented in academic databases. Reference lists of key included articles were also screened by hand to identify additional relevant studies.

### 2.3 Eligibility criteria and evidence taxonomy

Eligibility criteria were defined *a priori* to reduce selection bias and improve reproducibility. The review included primary empirical research, diagnostic validation studies, and formal policy or governance analyses that addressed AI, digital health, or stroke-care implementation in LMICs. Studies conducted in high-income settings were included only if they explicitly modeled technological translation to resource-constrained environments, such as lightweight algorithms, portable imaging, or decentralized deployment architectures. Editorials, expert commentaries, unreviewed preprints, and single-patient case reports were excluded. The search was limited to full-text articles available in English.

Because the included literature was intentionally heterogeneous, the evidence was classified into six categories: (1) empirical clinical studies, (2) diagnostic validation studies, (3) implementation and workflow studies, (4) policy and governance analyses, (5) technical or methodological papers, and (6) review articles. This taxonomy was used to ensure that studies were interpreted in relation to their evidentiary role rather than treated as a homogeneous set of clinical trials.

For empirical, validation, and implementation studies, the data charting template captured country/region, healthcare setting, stroke-care phase, primary data modality, study design, principal findings, and implementation limitations. For policy, standards, and review articles, the charting template captured scope, governance relevance, infrastructure implications, and contribution to the proposed framework. This structured approach allowed the review to summarize both direct empirical evidence and supporting contextual literature in a transparent and reproducible manner.

### 2.4 Study selection and data charting

Study selection was conducted in duplicate by two independent reviewers (X.J. and Y.H.). Titles and abstracts were screened first, followed by full-text evaluation of potentially eligible reports. Any disagreement between the two reviewers was resolved through discussion, and unresolved cases were adjudicated by a third senior reviewer (Z.Y.). This process was applied consistently across all eligible records to ensure objective selection of the final evidence set.

A structured data charting form was used to extract information from the included literature according to the evidence taxonomy described above. The charted variables were then synthesized narratively to identify recurring patterns across stroke-care phases, data modalities, implementation contexts, and governance challenges.

### 2.5 Methodological quality appraisal

To avoid overstating the strength of evidence, the methodological quality of primary empirical and diagnostic validation studies was appraised using the Mixed Methods Appraisal Tool (MMAT), 2018 version. The MMAT was selected because the included empirical literature comprised heterogeneous study designs, including randomized, non-randomized, observational, and mixed-methods studies. Assessment focused on selection bias, measurement validity, completeness of outcome data, and control of confounding where applicable.

Because policy papers, standards documents, and review articles do not conform to a single empirical design, they were not assigned MMAT scores. Instead, these sources were retained as contextual and conceptual evidence to support interpretation of implementation barriers, interoperability needs, and regulatory considerations. The appraisal results were used to contextualize the narrative synthesis rather than to exclude studies *post hoc*.

## 3 The structural crisis of stroke care in LMICs: a triad of systemic friction

The effectiveness of contemporary acute stroke care depends on a highly coordinated and time-sensitive continuum of care. In low- and middle-income countries (LMICs), however, this continuum is disrupted not by isolated deficiencies but by a set of interrelated structural constraints: limitations in clinical capability, restricted access to diagnostic imaging, and fragmentation of prehospital systems. Rather than producing linear delays, these factors interact to create a form of “temporal compression,” whereby therapeutic windows are substantially narrowed or missed entirely for a large proportion of patients (13). This systemic friction is particularly alarming given that LMICs now account for nearly 89% of global disability-adjusted life years (DALYs) lost to stroke, with projections from the WSO-Lancet Neurology Commission estimating a 50% increase in global stroke mortality to 9.7 million annually by 2050 if current trends persist (14).

### 3.1 Limitations in clinical capability: beyond workforce shortages

While the shortage of neurologists in LMICs is well-documented, the challenge extends beyond workforce numbers to encompass broader limitations in clinical capability across the entire care pathway. The workforce disparity is staggering; for instance, sub-Saharan Africa has only three neurologists per 10 million people, compared to up to 900 per 10 million in Europe—a 300-fold difference (15). Consequently, acute stroke care is frequently delivered by non-specialist providers in general medical wards, with limited access to standardized stroke training or structured assessment tools such as the NIH Stroke Scale (16).

These constraints heavily influence clinical decision-making, particularly concerning the use of thrombolytic therapy. Reports from LMIC implementation studies indicate that intravenous thrombolysis (IVT) rates remain consistently low. This is driven not only by late patient presentation but also by physician apprehension regarding hemorrhagic complications in environments lacking adequate monitoring capacity, intensive care beds, or trained stroke nurses (17, 18). Beyond the hyperacute phase, limitations in rehabilitation services represent a critical gap. Multidisciplinary rehabilitation teams are often unavailable or concentrated exclusively in urban tertiary centers, contributing to a poorer long-term functional outcome (19). To combat this capacity crisis, regional initiatives such as the African Stroke Organization's educational framework aim to train 5,000 stroke professionals by 2030, signaling a necessary shift toward structured workforce empowerment (20).

### 3.2 Restricted access to diagnostic imaging: the thrombolysis constraint

These clinical and workforce challenges are inextricably linked to restricted access to neuroimaging, creating a secondary bottleneck. Current stroke management guidelines strictly require brain imaging—typically non-contrast computed tomography (CT)—prior to initiating reperfusion therapy. However, in many LMICs, a profound “diagnostic void” severely limits this necessary step (21, 22). Available data reveal a catastrophic deficit: while high-income countries like Denmark boast nearly 44 CT scanners per million population, many low-income countries report fewer than one scanner per million (23). This hardware scarcity translates into severe clinical consequences. Imaging rates among stroke patients remain abysmally low, which directly correlates with poorer survival; notably, the 3-month stroke mortality rate is reported to be 4.5 times higher in LMICs than in high-income nations (7.7 vs. 1.7%) (24). Even when imaging hardware is physically available, out-of-pocket costs represent a substantial barrier for patients. Operational challenges further constrain effective access, with frequent interruptions due to equipment downtime, lack of maintenance infrastructure, and unreliable electricity supplies (25). Together, these factors paralyze timely diagnostic confirmation and drastically reduce eligibility for time-sensitive interventions.

### 3.3 Prehospital system fragmentation: delays in care pathways

Upstream of facility-based imaging and clinical care, limitations in prehospital systems represent an additional, often insurmountable barrier in the “first mile” of the stroke pathway. In many LMICs, formal emergency medical services (EMS) are underdeveloped, unevenly distributed, or vastly underutilized. A persistent lack of transportation infrastructure means that patients with stroke symptoms often bypass formal EMS entirely, relying instead on private transport, taxis, informal community networks, or even animal-drawn carts in remote rural settings (26, 27).

Recent global reviews and scientific statements from the World Stroke Organization highlight that delays in symptom recognition by the public, the lack of centralized EMS dispatch systems, and the absence of prehospital stroke triage protocols are ubiquitous systemic gaps (28, 29). A granular study from India, for example, directly identified the lack of symptom recognition and ensuing transport issues as the primary drivers of onset-to-door delays (30). These factors contribute to exponentially prolonged onset-to-door times. Furthermore, the absence of an organized EMS network eliminates the possibility of prehospital notification. Without advance communication from the field, emergency departments cannot pre-activate stroke pathways or reserve CT scanners, leading to compounding in-hospital delays for specialist consultation and imaging.

### 3.4 Systemic integration and policy imperatives

Taken together, these fragmented prehospital networks, diagnostic gaps, and shortages in the clinical workforce create a self-reinforcing cycle of system inefficiency (31, 32). Delayed presentation reduces eligibility for time-sensitive therapies; limited access to neuroimaging constrains evidence-based decision-making; and insufficient specialist capacity undermines both acute management and long-term rehabilitation (33).

From a health systems perspective, these interdependencies highlight the critical importance of strengthening the entire continuum of stroke care. Global strategies, including the WHO Intersectoral Global Action Plan on neurological disorders and the WSO–Lancet Neurology Commission, emphasize coordinated action across surveillance, prevention, acute treatment, and rehabilitation (34, 35).

Policy-oriented approaches increasingly advocate for comprehensive system-level reforms, including task-sharing models to extend care delivery, the development of telestroke networks to bridge specialist gaps, investment in diagnostic infrastructure, and optimization of emergency medical services through prehospital triage and notification systems (36, 37). Such coordinated and policy-driven interventions are essential to overcoming structural barriers and improving stroke outcomes in low- and middle-income countries.

## 4 A localized AI framework for stroke care: bypassing the temporal compression

Historically, advanced artificial intelligence (AI) architectures have been predominantly designed for resource-rich, high-income settings, relying heavily on ubiquitous high-speed cloud infrastructure and centralized data trusts (38). Transplanting these centralized paradigms directly into low- and middle-income countries (LMICs) inevitably fails. In these settings, severe power grid instability, profound data heterogeneity, and fragmented health system governance render cloud-dependent models highly brittle and clinically impractical (6).

Central to overcoming these barriers is addressing what we term “temporal compression.” In this framework, temporal compression is operationally defined not merely as a standard logistical metric (such as onset-to-needle time), but as a systemic mechanism whereby cumulative infrastructural, diagnostic, and cognitive delays progressively narrow and exhaust the viable therapeutic window for acute stroke intervention. To bypass this destructive mechanism, we must move beyond techno-solutionism. Instead, we propose a framework based on the rigorous adaptation and repurposing of decentralized technologies—specifically lightweight models, edge computing, and federated learning. By deliberately constraining these technologies to fit LMIC realities, we aim to embed resilient clinical utility directly into the stroke care continuum.

### 4.1 Lightweight model architectures: confronting hardware trade-offs

The foundation of this localized framework relies on lightweight model architectures. Originally pioneered for commercial mobile devices and Internet of Things (IoT) applications, techniques such as model quantization, pruning, and hybrid feature extraction are now being repurposed for frugal healthcare settings (39, 40). In LMIC district hospitals, edge devices typically operate under severe constraints regarding CPU capacity and thermal management. However, repurposing these commercial architectures for high-stakes clinical diagnostics requires confronting stark trade-offs. The capabilities of such models must be appraised with strict empirical objectivity. For instance, while advanced hybrid architectures (such as ViT-BiLSTM models) have achieved state-of-the-art diagnostic accuracies in MRI-based stroke classification, these models inherently contain over 10 million parameters (41). Achieving the ultra-fast inference times (e.g., below 20 ms) often touted in literature heavily relies on enterprise-grade GPU acceleration (such as NVIDIA T4 GPUs) rather than the standard, non-accelerated CPUs actually available in rural clinics (41). Therefore, deploying computer vision tasks in these settings requires acknowledging a deliberate compromise: sacrificing absolute computational speed for offline accessibility. Conversely, ultra-lightweight approaches, such as TinyML-based wearable sensors, demonstrate that when expectations are appropriately scaled, computational frugality can successfully drive continuous remote monitoring for post-stroke rehabilitation (42).

### 4.2 Edge computing: prioritizing resilience over latency

Edge computing was initially developed to minimize latency for bandwidth-intensive commercial applications like autonomous vehicles and 5G telecommunications. However, when adapted for LMIC stroke care, the primary value of edge computing is not necessarily achieving sub-millisecond speeds, but rather establishing infrastructural resilience (43).

In conventional digital health models, transmitting massive neuroimaging datasets (e.g., DICOM files) to centralized cloud servers introduces clinically unacceptable delays in rural regions plagued by intermittent connectivity (44, 45). Cloud dependence also exposes stroke pathways to systemic vulnerabilities during frequent power outages (46).

By relocating inference capabilities directly to the point of care—such as district hospitals or mobile diagnostic units—edge computing inherently acts as a buffer against network failures. In the context of acute stroke, where differentiating between ischemic and hemorrhagic events dictates immediate therapeutic pathways, this localized, offline processing capacity mitigates the effects of temporal compression (47). It ensures that basic, life-saving triage can proceed independently of the broader telecommunications grid.

### 4.3 Federated learning: navigating data sovereignty and fragmentation

Federated learning (FL) was initially conceptualized in high-income regions to navigate strict data privacy regulations (e.g., GDPR) by enabling decentralized model training without exchanging raw data (48). In LMICs, however, the utility of FL shifts toward solving a different problem: profound institutional fragmentation and the absence of centralized data trusts.

By allowing algorithms to be trained locally with only aggregated model updates shared across participating sites, FL provides a pathway for strengthening system-wide learning across under-resourced hospitals that may lack the governance capacity to legally or securely share raw patient data (49). It accommodates environments with intermittent connectivity, as local model training can proceed independently and synchronize only when network conditions permit (50). Yet, applying FL in LMICs introduces its own structural paradox. FL inherently relies on the availability of structured, digitized Electronic Health Records (EHRs). In health systems where paper-based records still predominate, the prerequisite digitization step remains a formidable barrier. Furthermore, decentralized training processes remain vulnerable to data poisoning or inference attacks, necessitating rigorous integration of differential privacy safeguards (51). Thus, FL should be viewed as a long-term architectural goal for LMICs, rather than an immediate plug-and-play solution.

### 4.4 Bridging technology and implementation science

While the repurposing of lightweight models, edge computing, and federated learning establishes a resilient technological infrastructure, technology alone cannot independently overcome the structural deficits of LMIC healthcare. A purely technical architecture risks falling into the trap of “pilotitis”—creating isolated digital solutions that function in controlled testing but fail to scale or integrate into real-world, underfunded medical practice.

To transition from theoretical potential to sustainable clinical impact, these repurposed digital tools must be operationalized through established implementation science theories and embedded within national health policies. Therefore, Section 5 shifts the focus from the technological “what” to the implementation “how.” By anchoring this localized AI approach in the Consolidated Framework for Implementation Research (CFIR), the subsequent section provides a prescriptive, actionable roadmap demonstrating exactly how this decentralized infrastructure must be adapted to the clinical workflows, governance structures, and policy imperatives of the stroke care continuum.

## 5 Re-engineering the stroke care continuum: a prescriptive implementation framework

To move beyond a descriptive synthesis of existing technologies, the “Localized AI + Policy” framework is explicitly designed as a prescriptive and theoretical roadmap for systemic implementation. It is crucial to position this framework as a direct technological continuation of the foundational implementation science established by seminal global stroke commissions.

Specifically, early implementation research by Pandian et al. (13) demonstrated that overcoming LMIC constraints requires moving beyond Western-centric, resource-heavy stroke unit models. They advocated instead for the rigorous reorganization of existing infrastructure through task-sharing and the establishment of regional “hub-and-spoke” networks. Concurrently, Owolabi, Feigin, and the WSO-Lancet Neurology Commission (14, 33) defined the “stroke quadrangle” (surveillance, prevention, acute care, and rehabilitation) and issued an urgent call for pragmatic, system-integrated solutions to address the projected 50% increase in global stroke mortality by 2050.

**Figure 3:**
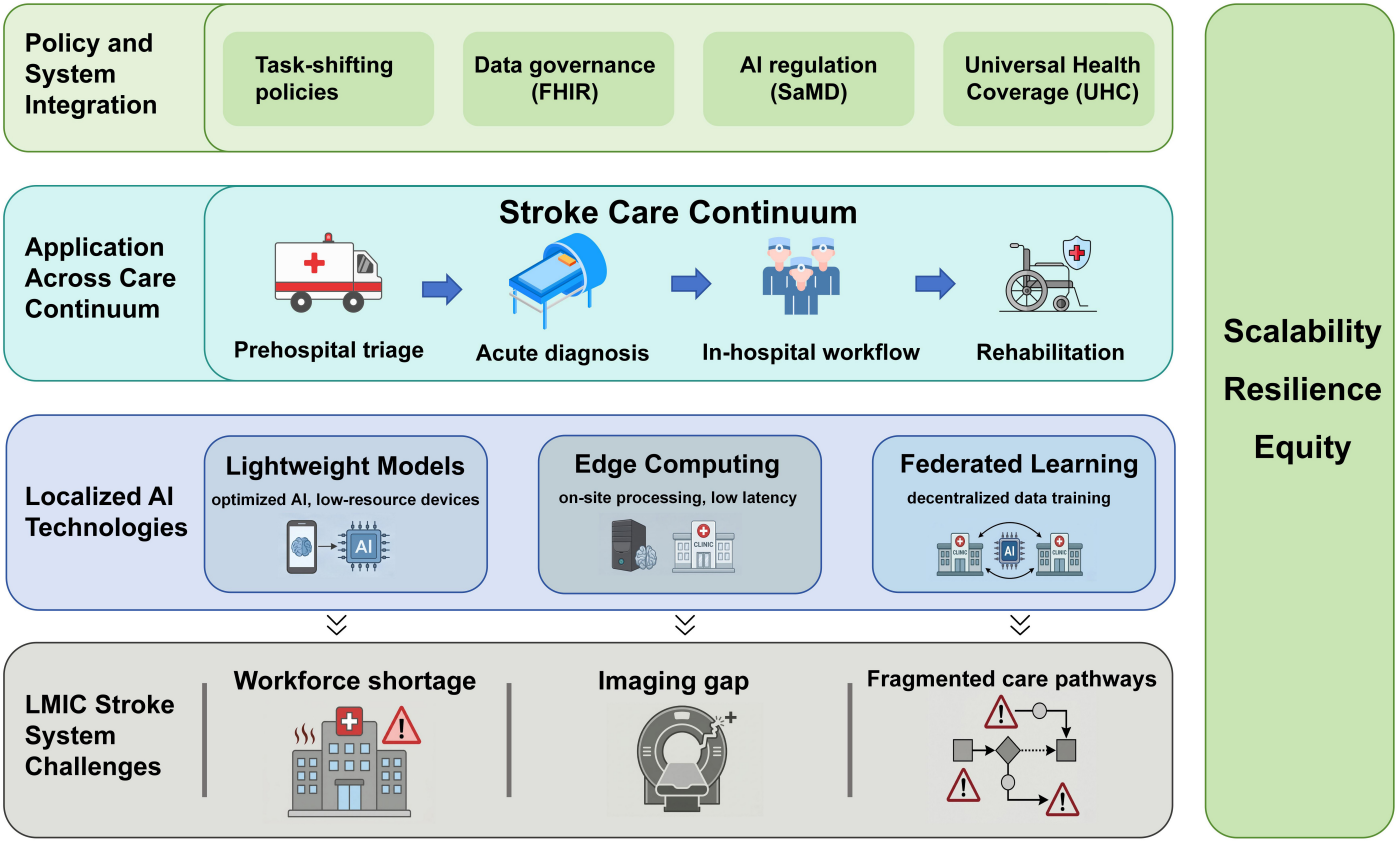
Proposed localized AI and policy integration framework for reducing stroke care inequity in LMICs, illustrating the synergistic interaction between resource-adapted AI technologies and health system governance mechanisms across the stroke care continuum. As indicated by the qualitative mapping arrows, each technological pillar directly addresses a specific LMIC structural barrier: Lightweight Models mitigate workforce shortages by enabling offline, task-shifted prehospital triage; Edge Computing overcomes diagnostic imaging gaps by bypassing cloud latency to deliver on-site acute inference; and Federated Learning addresses fragmented care pathways by integrating isolated clinical data silos without compromising local data sovereignty.

Our research explicitly builds upon their theoretical foundations. While these seminal works outlined what systemic reorganizations are structurally required (e.g., task-shifting, prehospital triage), our framework provides the decentralized digital architecture required to operationally execute them. By mapping this continuity against the Consolidated Framework for Implementation Research (CFIR) (52) and the WHO Health Systems Building Blocks (53), our framework illustrates how localized AI functions as a catalyst to combat temporal compression (Figure 3). To contextualize this approach, the following subsections critically explore how these technologies intersect with recent initiatives across various LMIC settings, culminating in measurable criteria for evaluating policy alignment.

### 5.1 Prehospital triage and task-shifting: empowering community health workers

Addressing the WHO building block of the “Health Workforce” requires leveraging existing community structures (CFIR Outer Setting). As advocated by global stroke guidelines, task-sharing is paramount in regions where formal Emergency Medical Services (EMS) are severely underdeveloped. Rwanda's globally recognized Community Health Worker (CHW) program exemplifies a robust structural alternative, deploying over 45,000 CHWs who serve as the critical first point of contact in primary care (54).

Recently, the Rwanda Biomedical Center and PATH launched prospective observational diagnostic accuracy studies (“silent trials”) to evaluate how Generative AI assistants can guide CHWs through complex diagnostic protocols (55). Applying our framework to stroke triage, offline lightweight AI can theoretically be deployed directly onto CHW smartphones. However, the hardware reality imposes strict functional limitations. For instance, while advanced lightweight vision models like TE-YOLOv5 have achieved impressive precision (81.5%) in delineating stroke lesions, their touted ultra-fast inference typically relies on GPU acceleration (56). Additionally, while AI-enhanced portable low-field MRI (LF-MRI) systems present a promising alternative to heavy diagnostic infrastructure in resource-depleted settings (57), building on prototype ultra-low-field scanners that have already demonstrated point-of-care brain imaging using a standard AC power outlet (58, 59), their lower resolution mandates caution. Therefore, on consumer-grade mobile devices, these algorithms must be strictly defined and regulated as risk-screening tools designed solely to optimize referral pathways, rather than functioning as standalone diagnostic devices.

### 5.2 Acute phase workflow orchestration: confronting infrastructure realities

Upon facility arrival, the challenge shifts to the CFIR “Inner Setting”—specifically, the digital networks required for rapid intervention. Historically, large LMICs like Brazil have struggled with profound “care deserts” in regions lacking specialist infrastructure. To address this disparity, recent macro-initiatives have aggressively modernized the Unified Health System (SUS). In July 2025, Brazil's Decree No. 12,560/2025 launched the National Health Data Network (RNDS) to mandate interoperability across the country. Concurrently, the Brazilian Artificial Intelligence Plan (PBIA) 2024–2028 has committed an estimated $ 23 billion in investments, specifically targeting “Diagnostic Optimization in the SUS” for critical conditions like stroke (60).

Within our framework, edge computing acts as the critical bridge to operationalize these national policies at the local level. Validated by Brazil's RESILIENT-Extend public health trials—which demonstrated the viability of selecting patients for thrombectomy without expensive perfusion imaging (61)—on-premise edge nodes can be deployed at district hospitals to rapidly identify highly suspicious scans. This localized processing triggers prioritized alerts across the RNDS, ensuring the “parallel mobilization” of remote specialists. This approach aims to replicate the workflow efficiencies of high-income models, which have demonstrated up to 39.5-min reductions in notification times via AI coordination (62). A systematic review further corroborates this trend, reporting that AI-based large vessel occlusion detection tools can save an average of 52 min to treatment, reinforcing the potential of AI as a workflow orchestrator rather than a standalone diagnostic (63).

However, to maintain scientific rigor, the limitations of current AI models must be explicitly acknowledged. While AI-assisted non-contrast CT (NCCT) interpretation has demonstrated improvements in non-specialist diagnostic sensitivity—from 25.4 to 33.3% in a multicenter study (64), and from 33.8 to 53.7% with deep learning-assisted ASPECTS evaluation (65)—these absolute performance levels remain clinically insufficient and carry a substantial risk of false negatives. Accordingly, such tools should be regarded primarily as workflow-support systems rather than standalone diagnostic solutions. Similarly, reported workflow gains, including a 30.2-min reduction in door-to-treatment time with AI-assisted triage (66), should be interpreted cautiously, as single-center before-and-after studies remain vulnerable to secular trends, Hawthorne effects, and concurrent process improvements.

Importantly, the narrative that edge computing unconditionally resolves LMIC infrastructure deficits requires critical qualification. While edge nodes successfully relax high-bandwidth dependencies, they remain fundamentally tethered to consistent electrical power. This represents a profound implementation barrier, given that a 2023 WHO assessment indicates up to 41% of healthcare facilities in regions like Sub-Saharan Africa lack reliable access to electricity grids (67). Sustainable edge AI deployments must therefore be strictly coupled with localized Uninterruptible Power Supply (UPS) or solar-backed infrastructure to prevent hardware failures and ensure clinical reliability during inevitable grid outages. Further emphasizing the need for systemic integration, the Nepal Stroke Project demonstrated that dedicated stroke services in a lower-middle-income country could be elevated from minimal to essential levels within 18 months through a combination of healthcare professional training, in-hospital quality monitoring, and public awareness campaigns; however, the project also identified persistent barriers around government ownership and financial sustainability that must be addressed for long-term success (68).

### 5.3 Cognitive task-shifting: the health economics of decentralized triage

Beyond perceptual imaging tasks, the framework must address the cognitive bottleneck of triage in deep rural settings (WHO “Service Delivery” block). In countries like India, experiments with physical Mobile Stroke Units (MSUs) have demonstrated excellent clinical efficacy by bringing the hospital to the patient (69). Yet, they face insurmountable scale-up barriers due to prohibitive costs. Recent global evaluations indicate that the capital expenditure for a single MSU ranges from $0.7 to $1.8 million USD, alongside median annual operational costs of $1.0 million USD, rendering them economically unsustainable for widespread public deployment in LMICs (70). In stark contrast, deploying edge-capable AI servers—typically costing under $10,000 USD per node, with inference costs representing fractions of a cent—presents a drastically more viable and scalable health economic model for decentralized triage (71). This aligns with health-economic evidence that telestroke networks, another form of decentralized care, can be a dominant, cost-saving strategy in middle-income settings (72).

Furthermore, rural district clinics, as highlighted by population-based registries such as the Ludhiana Stroke Registry, frequently lack the specialized personnel required to initiate acute therapies safely (73). To facilitate genuine task-shifting in these clinics, the framework prescribes the integration of Retrieval-Augmented Generation (RAG) and Knowledge Graph-based Clinical Decision Support Systems (CDSS) (74, 75). By grounding generic Large Language Models exclusively in codified EHR data and verified national guidelines, RAG architectures mitigate the risks of AI hallucinations. This provides frontline general practitioners with transparent, step-by-step thrombolysis decision pathways, empowering them with the cognitive support necessary to safely execute time-critical interventions locally. The real-world potential of such decision support is underscored by a landmark LMIC trial, in which an AI-based CDSS reduced new vascular events by 26% at 3 months (76).

### 5.4 Post-acute interoperability: the paradox of federated learning

Finally, the framework confronts the fragmentation of longitudinal secondary prevention (WHO “Health Information Systems” block). Genuine data liquidity across the care continuum requires governance by modern interoperability protocols, such as the Fast Healthcare Interoperability Resources (FHIR) guidelines—specifically the Stroke FHIR Implementation Guide incorporating ICHOM patient-centered outcome sets (77).

Building upon this interoperable foundation, the framework leverages Federated Learning (FL). FL enables distributed LMIC hospital networks to collaboratively train predictive models for secondary prevention without centralizing sensitive patient data (78). Furthermore, highly optimized hybrid architectures can be integrated into these federated frameworks to continually refine MRI-based stroke classification while strictly preserving local data privacy (41).

However, applying FL in LMICs introduces a structural paradox that is often overlooked in algorithmic literature. FL inherently relies on the availability of high-quality, structured Electronic Health Records (EHRs). In many district hospitals where paper-based documentation still predominates, the very sites that most desperately need decentralized AI lack the digital prerequisites to participate in it (79). Bridging this digital divide requires the preliminary implementation of low-cost Optical Character Recognition (OCR) tools or structured mobile data-entry applications. Without these foundational digitization steps, federated algorithms cannot meaningfully converge on heterogeneous data ecosystems. This phased approach aligns directly with the WHO/ITU Global Initiative on AI for Health (GI-AI4H) mandates for privacy-preserving and standardized AI architectures (80).

### 5.5 Operational criteria for policy alignment and trustworthy AI

To ensure the “Localized AI + Policy” framework is actionable rather than merely rhetorical, it is necessary to establish operational definitions. “Alignment with national policy frameworks” is operationally defined as the extent to which an AI deployment integrates into the formal regulatory, financial, and clinical governance structures of the host system (6).

Drawing upon the FUTURE-AI consensus guidelines (81) and operational AI governance checklists (82), specific deployments conform to this framework only if they satisfy the following critical criteria:

Algorithmic Equity and External Validity: The vast majority of clinical AI algorithms are trained on geographically homogeneous cohorts from high-income regions (e.g., just three US states dominate training datasets) (83). Deployments must mandate local recalibration and fairness audits to prevent algorithmic bias from exacerbating health disparities when applied to LMIC populations with differing imaging protocols and demographics, as established by Chen et al. (84).

Post-deployment monitoring for model drift: Offline edge AI systems are highly susceptible to “model drift” over time as clinical protocols or local population characteristics evolve. Frameworks must establish clear financial and governance models for continuous maintenance, including periodic online synchronization, safety signal detection, and model retraining to ensure sustained clinical accuracy (85).

Regulatory conformance (Governance): The AI system must be classified and regulated as Software as a Medical Device (SaMD) under national regulatory frameworks and undergo clinical validation reflecting local demographic data (86).

Workflow and accountability (Workforce): The AI must not operate autonomously. The deployment requires documented “human-in-the-loop” protocols embedded in formal clinical pathways, ensuring clear medico-legal liability is retained by a designated clinical entity.

Financial integration (Financing): The intervention must transition beyond temporary pilot grant funding to be formally integrated into the operational budgets of national health programs or covered under Universal Health Coverage (UHC) reimbursement schemes.

## 6 Discussion: scope, alternative paradigms, and governance

While Sections 4 and 5 established the technological feasibility and implementation roadmap, this section critically evaluates the framework's clinical scope, addresses the inherent limitations of decentralized data architectures, and outlines the regulatory frontiers necessary for sustainable deployment.

### 6.1 Clinical scope, usability, and stroke disparities

A primary critique of digital health interventions in LMICs is their ambiguity regarding practical utilization. The proposed framework is deliberately designed for multi-stakeholder operationalization: (1) researchers and hospital IT staff orchestrate decentralized edge pipelines; (2) clinical providers—ranging from community health workers to general practitioners—interact with AI-assisted decision systems; and (3) public health administrators govern scaling and reimbursement strategies.

Clinically, the scope of this framework spans the entire stroke continuum, from pre-hospital triage to acute imaging workflows and post-acute rehabilitation, reflecting established stroke care pathways (87). Furthermore, while stroke-related disparities persist globally, LMIC health systems face disproportionate constraints in infrastructure, workforce capacity, and resource allocation (88).

### 6.2 Rethinking data architectures: federated learning vs. traveling models

While Federated Learning (FL) preserves data sovereignty, it does not inherently resolve data heterogeneity. In highly variable LMIC settings, FL introduces challenges including non-IID data distributions, schema inconsistencies across electronic health record (EHR) systems, and unequal computational resources across institutions (89). These factors can degrade model performance and complicate convergence in distributed learning systems (90).

Additionally, FL requires frequent communication and synchronization across nodes, introducing significant communication overhead and dependency on stable network infrastructure. These constraints are particularly problematic in under-resourced settings.

To address these limitations, alternative paradigms such as the “Model-to-Data” or Traveling Model (TM) approach have been proposed. In contrast to synchronous aggregation in FL, sequential model transfer can reduce communication overhead and improve robustness in heterogeneous environments.

Furthermore, future frameworks must evolve beyond unimodal neuroimaging. The integration of multimodal data—including imaging, clinical variables, and structured EHR data—has been shown to enhance predictive performance and improve clinical decision-making accuracy.

### 6.3 Adaptive regulatory pathways and trustworthy AI

Alignment with national policy frameworks requires operational definitions grounded in existing governance standards. Drawing upon international guidelines for trustworthy AI, deployments must satisfy several critical criteria.

Algorithmic equity: clinical AI systems are frequently trained on geographically homogeneous datasets from high-income regions, limiting generalizability. Local recalibration is therefore necessary to mitigate bias when deploying models in LMIC populations.

Model drift monitoring: distributed and edge-based AI systems are susceptible to performance degradation over time due to evolving data distributions, necessitating continuous monitoring and retraining strategies.

Adaptive regulatory pathways: traditional regulatory models are insufficient for continuously learning AI systems. Instead, total product lifecycle (TPLC) approaches emphasize ongoing monitoring, evaluation, and iterative updates throughout deployment.

Workflow and accountability: AI systems must remain human-in-the-loop, ensuring that clinical responsibility and decision-making authority remain with healthcare professionals.

Financial integration: sustainable implementation requires integration into national health financing mechanisms, consistent with global digital health strategies emphasizing system-level adoption and governance.

## 7 Conclusion

Reducing the escalating global burden of stroke in LMICs demands a fundamental systemic reconfiguration. Overcoming “temporal compression” requires a transition from centralized techno-solutionism to a decentralized, resilient architecture. By integrating lightweight models, edge computing, and adaptive data paradigms (such as Traveling Models and multimodal integration) into national health policies, the “Localized AI + Policy” framework provides a pragmatic roadmap. Ultimately, realizing equitable stroke care depends on the capacity of LMIC health systems to embed these frugal innovations within robust strategies for digital sovereignty, task-shifting, and adaptive regulatory governance.
