# Supplementary material for: Localized AI for stroke care in LMICs: a framework to overcome structural and diagnostic barriers
Source: Front Public Health. 2026 Jun 10;14:1859276. doi: 10.3389/fpubh.2026.1859276 (PMC13290767; doi:10.3389/fpubh.2026.1859276)
Supplement: Supplementary file 3 [file Data_Sheet_1.pdf]

Supplementary Material 1: Detailed Database Search Strategies

Database	Search Strategy	Filters / Limits
**PubMed**	(("Stroke"[Mesh] OR "Brain Ischemia"[Mesh] OR stroke*[tiab] OR cerebrovascular[tiab] OR "cerebrovascular disease*"[tiab] OR "intracerebral hemorrhage"[tiab] OR "ischaemic stroke"[tiab] OR "ischemic stroke"[tiab]) AND ("Artificial Intelligence"[Mesh] OR "Machine Learning"[Mesh] OR "artificial intelligence"[tiab] OR AI[tiab] OR "machine learning"[tiab] OR "deep learning"[tiab] OR "neural network*"[tiab] OR "computer vision"[tiab] OR "clinical decision support"[tiab] OR "edge computing"[tiab] OR "federated learning"[tiab] OR "digital health"[tiab] OR telehealth[tiab] OR telemedicine[tiab] OR telestroke[tiab]) AND ("Developing Countries"[Mesh] OR "low- and middle-income countr*"[tiab] OR LMIC*[tiab] OR "resource-limited"[tiab] OR "resource-constrained"[tiab] OR "low-resource"[tiab] OR Afghanistan[tiab] OR Bangladesh[tiab] OR Benin[tiab] OR Bolivia[tiab] OR Cameroon[tiab] OR Egypt[tiab] OR Ethiopia[tiab] OR Ghana[tiab] OR India[tiab] OR Indonesia[tiab] OR Kenya[tiab] OR Nepal[tiab] OR Nigeria[tiab] OR Pakistan[tiab] OR Peru[tiab] OR Philippines[tiab] OR Rwanda[tiab] OR Senegal[tiab] OR "South Africa"[tiab] OR Sri Lanka[tiab] OR Tanzania[tiab] OR Uganda[tiab] OR Ukraine[tiab] OR Vietnam[tiab] OR Zambia[tiab] OR Zimbabwe[tiab])) AND (2015:2026[pdat]) AND english[lang]	**Date:** January 1, 2015 — March 31, 2026<br>**Language:** English<br>**Species:** Humans

**Google Scholar**	`("stroke" OR "cerebrovascular disease" OR "brain ischemia") AND ("low- and middle-income countries" OR "LMICs" OR "resource-constrained" OR "global south") AND ("artificial intelligence" OR "machine learning" OR "edge computing" OR "federated learning" OR "digital health")`	**Date:** 2015—2026 *Note: Searched using Publish or Perish software to extract top 200 relevance-ranked results per keyword combination due to Google Scholar's lack of Boolean precision and large volume.*

**IEEE Xplore**	(("All Metadata":"stroke" OR "All Metadata":"cerebrovascular" OR "All Metadata":"brain ischemia" OR "All Metadata":"intracerebral hemorrhage") AND ("All Metadata":"artificial intelligence" OR "All Metadata":"machine learning" OR "All Metadata":"deep learning" OR "All Metadata":"neural network" OR "All Metadata":"edge computing" OR "All Metadata":"federated learning" OR "All Metadata":"digital health" OR "All Metadata":"telemedicine" OR "All Metadata":"telestroke" OR "All Metadata":"clinical decision support") AND ("All Metadata":"low– and middle–income countries" OR "All Metadata":"LMIC" OR "All Metadata":"resource–limited" OR "All Metadata":"resource–constrained" OR "All Metadata":"low–resource" OR "All Metadata":"India" OR "All Metadata":"Nigeria" OR "All Metadata":"Kenya" OR "All Metadata":"Pakistan" OR "All Metadata":"Bangladesh" OR "All Metadata":"Nepal" OR "All Metadata":"Ethiopia" OR "All Metadata":"Ghana" OR "All Metadata":"Uganda" OR "All Metadata":"Tanzania"))	**Date:** 2015—2026<br>**Content Types:** Journals, Early Access Articles, Conference Proceedings<br>**Language:** English

Abbreviations: MeSH = Medical Subject Headings; TS = Topic Search; LMICs = Low– and Middle–Income Countries.
